# Urethral Sphincter Botulinum Toxin A Injection for Non-Spinal Cord Injured Patients with Voiding Dysfunction without Anatomical Obstructions: Which Patients Benefit Most?

**DOI:** 10.3390/toxins15020087

**Published:** 2023-01-17

**Authors:** Sheng-Fu Chen, Hann-Chorng Kuo

**Affiliations:** 1 Department of Urology, Hualien Tzu Chi Hospital, Buddhist Tzu Chi Medical Foundation, Hualien 970, Taiwan; 2 Department of Urology, Buddhist Tzu Chi University, Hualien 970, Taiwan

**Keywords:** voiding dysfunction, detrusor underactivity, dysfunctional voiding, poor relaxation of the external sphincter, onabotulinum toxin A

## Abstract

Objective: Treating voiding dysfunction without anatomical obstructions is challenging. Urethral onabotulinum toxin A (BoNT-A) is used in treating voiding dysfunction; however, the success rate varies widely, and patients may not be satisfied with the treatment outcome. This study compared the efficacy of the urethral BoNT-A injection between patients with different non-spinal cord injury (SCI) voiding dysfunctions. Materials and Methods: This study retrospectively analyzed patients with refractory voiding dysfunction, including detrusor underactivity (DU), dysfunctional voiding (DV), and poor relaxation of the external sphincter (PRES) who received the urethral sphincter 100 U BoNT-A injection. The treatment outcomes were assessed via a global response assessment (GRA) one month after treatment. Baseline and follow-up videourodynamic study (VUDS) parameters were also compared. Results: Totally, 161 patients (60 with DU, 77 with DV, and 24 with PRES) with a mean age of 58.8 ± 20.2 were enrolled, of which 62.1% had a good response (GRA ≥ 2) after urethral BoNT-A injection. DV patients had a higher success rate (76.6%) than DU (50%) and PRES (45.8%) patients (*p* = 0.002). A diagnosis of DV, higher voided volume and recurrent urinary tract infection were predictors of a good treatment response, while the cervical cancer status post-radical surgery predicted a poor response. Receiver operating characteristic (ROC) curve analyses identified PVR > 250 mL as a negative predictor (*p* = 0.008) in DU patients. Conclusions: The urethral BoNT-A injection provides a satisfactory success rate for non-SCI voiding dysfunction. Patients with DV benefit most from both subjective and objective parameters. Approximately 50% of patients with DU and PRES also had a fair response. PVR > 250 mL was a negative predictor in DU patients.

## 1. Introduction

Neurogenic or non-neurogenic voiding dysfunction, with symptoms of difficulty voiding and large post-void residual (PVR) volumes, which may result in upper urinary tract deterioration if not well managed. Voiding dysfunction without neurogenic insult may be due to detrusor underactivity (DU) and bladder outlet obstruction (BOO, such as benign prostate hyperplasia, bladder neck dysfunction, or urethral sphincter hyperactivity, like dysfunctional voiding (DV) [1] or poor relaxation of the external urethral sphincter (PRES) during micturition [2].

DU is a common urological condition whose treatment has remained challenging. In a recent study, detrusor contractility may be reversed by the medical or surgical treatment [3]. Surgical techniques such as transurethral incision of the bladder neck (TUI-BN) or transurethral resection of the prostate (TUR-P) and urethral onabulinumtoxinA (BoNT-A) injection aim to decrease bladder outlet resistance [3]. Urethral injections of BoNT-A were first used for patients with detrusor sphincter dyssynergia or patients with spinal cord injury (SCI), and it effectively decreased the urethral pressure profile and PVR volume [4]. Phelan et al. confirmed the therapeutic efficacy of sphincteric BoNT-A injections in SCI patients with various etiologies of DSD in both men and women. BoNT-A decreases urethral resistance in pharmacology by paralyzing the striated sphincter muscle through the inhibition of acetylcholine release from the neuromuscular junction [5]. In recent years, some studies reported different causes of urethral sphincter dysfunction, be it neurogenic or non-neurogenic, and significant improvements in voiding after sphincteric BoNT-A injections [6].

Although urethral BoNT-A injections have been used in treating non-SCI patients with voiding dysfunction in recent years, the success rate varies widely, and patients could be unsatisfied with the outcome. A previous randomized, double-blind, placebo-controlled trial showed the success rate was not superior to that of normal saline injections [7]. We believe inducing powerful abdominal pressure by straining is necessary for voiding in DU patients; conversely, in patients with DV or PRES, adequate relaxation of the urethral resistance is needed to achieve efficient voiding. Therefore, some patients have benefits in terms of subjective or objective responses. Our previous study reported a 60% success rate in DU and voiding dysfunction after BoNT-A injections [8]. Nowadays, it still is an alternative treatment and not a standard one, and which of them benefits patients with voiding dysfunction most is unclear. We aimed to analyze the efficacy of urethral BoNT-A injections in treating voiding dysfunction in non-SCI patients and compare the therapeutic efficacy between the different etiologies of voiding dysfunction (DU, DV, and PRES).

## 2. Results

A total of 161 patients with voiding dysfunction refractory to medical therapy with a mean age of 58.8 ± 20.2 years who underwent urethral BoNT-A injection were enrolled. The patients were divided into three subgroups as follows: 60 DU patients (19 males and 41 females), 77 DV patients (10 males and 67 females), and 24 PRES patients (14 males and 10 females). The probable underlying comorbidities and bladder conditions that could be related to voiding dysfunction are listed in Table 1.

The VUDS characteristics before and after treatment in the three study groups were compared, and the results are listed in Table 2. In female patients with DV, P_det_.Q_max_ significantly decreased after urethral BoNT-A injection (from 60.1 ± 36.0 to 47.6 ± 32.6, *p* = 0.004), and bladder outlet obstruction indext (BOOI) also significantly decreased in female DV patients (from 48.2 ± 35.8 to 30.0 ± 33.5, *p* = 0.000). The changes in P_det_.Q_max_ and BOOI in female DV patients were also statistically significant compared with the DU and PRES groups (*p* = 0.000 and *p* = 0.002). Other videourodymamic parameters did not differ significantly after urethral BoNT-A injections.

Treatment outcomes, per the scaled GRA as described in the methodology, are listed in Table 3. The GRA was recorded a month after treatment. Per the postoperative GRA, we divided patients into three groups (0–1, 2, and 3). GRA ≥ 2 was considered a successful outcome. Overall, 100 of 161 (62.1%) non-SCI patients with voiding dysfunction were successfully treated using urethral BoNT-A injections. As shown in Table 3, younger patients responded better to treatment (*p* = 0.016). On the other hand, sex was not significantly associated with treatment outcomes (*p* = 0.127). Finally, among patients with different voiding dysfunctions, we found that DV patients had better treatment outcomes than those with DU and PRES (*p* = 0.002). Approximately 76.6% of DV patients reported GRA ≥ 2, while 50% of DU patients and 45.8% of PRES patients reported GRA ≥ 2. 64 patients were under CIC and baseline and 30 patients voided well without CIC.

**Figure 1:**
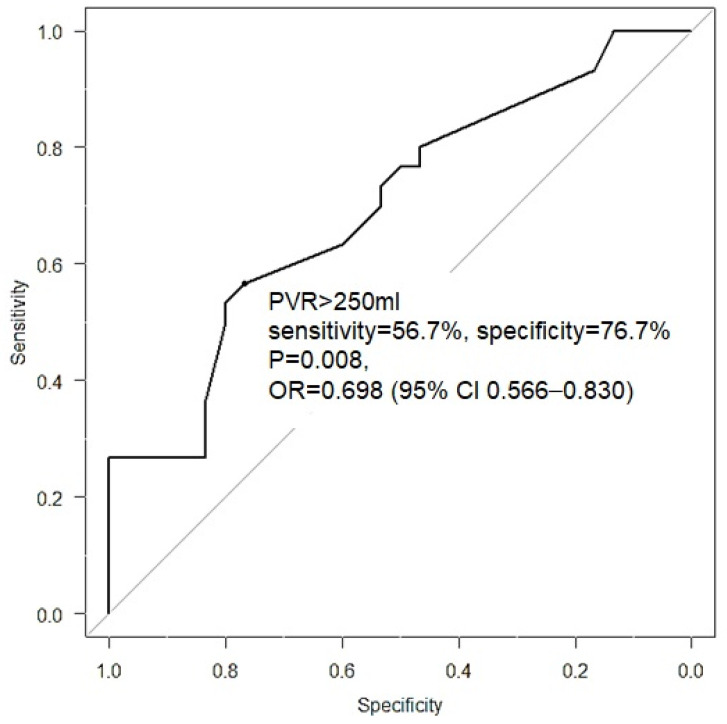
Receiver operating characteristic analysis of the baseline post-void residual (PVR) volume in patients with detrusor underactivity.

We searched the predictive factors related to the treatment outcome of the baseline characteristics, including the underlying disease, lower urinary tract condition, and VUDS parameters. During multivariate analyses of factors associated with GRA ≥ 2 in the treatment outcome of patients with non-SCI voiding dysfunction, a diagnosis of DV (OR = 3.630, *p* = 0.002), more voided volume (OR = 1.004, *p* = 0.014) at baseline, and a history of recurrent urinary tract infection (UTI) (OR = 3.949, *p* = 0.007) were predictors of good treatment response. On the other hand, cervical cancer was a predictor of a poor treatment outcome (OR = 0.214, *p* = 0.008) (Table 4). Because only approximately 50% of DU patients had satisfactory outcomes, we further analyzed which factors could be better indicators of a good response. We found that a large PVR was a negative predictive factor for DU patients (OR = 0.995, *p* = 0.011). Because PVR is a predictor of a poor outcome in DU patients, a ROC curve analysis was performed. Figure 1 shows that PVR > 250 mL is a negative predictive factor for urethral BoNT-A injection in DU patients (Sensitivity = 0.567, specificity = 0.767, *p* = 0.008).

## 3. Discussion

Per our findings, the BoNT-A urethral sphincter injection in non-SCI patients with voiding dysfunction produced a good response in 62.1% of patients after the urethral BoNT-A injection. In different types of voiding dysfunction, patients with DV had better treatment outcomes than those with DU and PRES. In multivariate analyses, DV, more voided volume, and recurrent UTI, were predictors of a good response to treatment, while the cervical cancer status post-radical surgery predicted a poor response. Although VUDS parameters did not differ significantly before and after treatment, they allow physicians to clearly observe the bladder outlet appearance during the voiding phase, which may provide insights into the pathophysiology of the voiding dysfunction [8]. We found that DV is a good predictor of the treatment response; so VUDS is considered to play an important role in making a precise diagnosis before treatment.

BoNT-A is believed to block the presynaptic release of acetylcholine in the neuromuscular junction in striated muscles, which achieves medical sphincterotomy effects. This could reduce the urethral sphincter resistance and improve voiding dysfunction [9]. The application of BoNT-A in urology was first used with urethral sphincter injections for the treatment of detrusor sphincter dyssynergia in patients with SCI and multiple sclerosis [10]. Double-blind placebo-controlled study then confirmed the validity and durability of the therapeutic efficacy of the BoNT-A urethral sphincter injection for patients with SCI and DSD [10]. Therefore, this treatment has been further used in treating non-SCI voiding dysfunction patients nowadays due to urethral sphincter hyperactivity, PRES, and DV or DU [5].

Voiding dysfunction is a frequently encountered clinical problem. In addition to anatomical obstruction-related voiding dysfunctions like benign prostatic hyperplasia and urethral stricture, functional problems like DU, DV, or PRES are more challenging for urologists. The current urodynamic study reported DU would possess in 12.4% of men [11] and 23.1% of women [12] with voiding dysfunction. Urethral sphincter hyperactivity was found in 17.0% of women, and PRES was noted in 39.5% of men and 17.6% of women with voiding dysfunction [11,13].

Treatment of DV is usually challenged because the actual pathophysiology has not been well explained currently and is thought to be a dysregulated urethral function with a spastic or non-relaxing external urethral sphincter during voiding [14]. DV results in difficult voiding and leads to a weak stream of urination and a large PVR. Therefore, attempts to reduce the hypertonicity or hyperactivity of the urethral sphincter via oral medication and resume smooth voiding are often futile. It is also postulated that voiding dysfunction due to psychological origins such as anxiety or depression might cause low detrusor contractility and urethral sphincter non-relaxation by inhibiting detrusor contraction [15]. Liao et al. previously reported an overall success rate of 86.7% for DV patients with sphincteric injections (50–100 units of Botox) [14]. Lee et al. also reported a 62.2% success rate in non-neurogenic DV [16]. In our study, approximately 76.6% of DV patients treated using BoNT-A urethral sphincter injections had a GRA of ≥2. VUDS also showed significantly decreased P_det_.Q_max_ and BOOI in female patients. On the other hand, our result showed no significant difference in male DV after treatment in VUDS data. It may be due to the case number being small (*n* = 10), and we also found that male DV baseline detrusor contractility is relatively not strong enough. 

The etiology of DU is known to be neurogenic, myogenic, obstructive, or idiopathic. Sustained abdominal pressure is necessary to facilitate emptying the bladder [17]. Urethral BoNT-A sphincter injections help to decrease bladder outlet resistance and achieve successful outcomes. We need to be sure that the bladder neck should open during voiding. Otherwise, BoNT-A injections to the urethral sphincter may not be successful [14]. Therefore, if bladder neck dysfunction was confirmed by VUDS, patients with DU and voiding dysfunction should receive transurethral incision of the bladder neck (TUIBN) rather than urethral BoNT-A injection. In this study, we carefully excluded patients with bladder neck dysfunction and those previously treated for TUIBN (73.9%). In this study, approximately 50% of DU patients had GRA ≥ 2. This means the recovery of detrusor function combined with a hyperactive sphincter also suggested the potential neuromodulatory effect of the sphincteric BoNT-A injection. Sufficient abdominal pressure is necessary for triggering spontaneous voiding after urethral BoNT-A injections in patients with DU. In our previous study, female DU patients exhibited VE improvement after active treatment, and intact bladder sensations and smaller PVR had better treatment outcomes [18]. In this study, we also found that a large PVR is a negative predictor, and the receiver operating characteristic curve showed that PVR > 250 mL at baseline indicates a poor outcome. We supposed that a large PVR indicates a lower abdominal pressure or decreased bladder sensation. Another important factor for efficient urination is acceptable bladder sensation. The sensory afferents from the bladder urothelium and detrusor play important roles in the voiding reflex circuit. Decreased bladder sensation will render the initiation of voiding difficult [19]. Overall, DU patients treated using BoNT-A urethral sphincter injections in our study showed a 50% success rate. We also found that female patients with cervical cancer status post-radical hysterectomy had poor outcomes. We believe radical surgery causes nerve injury and, thus, irreversible DU; so, we could predict that patients with poor sensations and large PVRs were usually not satisfied with the treatment.

PRES, as a diagnosis, was determined based on the voiding phase in the VUDS, which shows non-relaxed surface EMG activity combined with a narrow membranous urethra [2]. The etiology of PRES was considered multifactorial, such as potential neuropathy, learned habituation, pelvic floor hypertonicity, and bladder hypersensitivity [20]. PRES is characterized by relatively small but stable bladders and low-pressure/low-flow during the voiding phase [21], which is different from the typical high-pressure/low-flow presentation in DV. Urethral BoNT-A injections may provide benefits by inhibiting acetylcholine release in the neuromuscular junction to reduce urethral resistance. Because the typical PRES is low-pressure during the voiding phase, we supposed that there was also inadequate detrusor contractility. Therefore, the success rate of urethral BoNT-A injections is not as high as that of DV. On the other hand, a previous study showed that detrusor contractility might be restored after BoNT-A injections in DU patients with PRES [22]. This result supports the hypothesis that the low-pressure/low-flow dysfunction present in PRES might be the result of the detrusor suppression induced by non-relaxed urethral sphincter activity [7].

The primary limitation of this study is its retrospective design, different group sizes, and single-center scope of evaluation. Second, the 1–6-month follow-up VUDS was not consistent, which may have influenced the results of objective parameters. However, we believe the efficacy of BoNT-A durability continued for at least 6 months [23]. Moreover, patients without follow-up VUDS were not enrolled in this study, which might have caused selection bias. In this study, no obvious side was reported. However, we supposed mild side effects might exist in some patients, such as urinary incontinence. Finally, the patient groups were heterogeneous, with varying causes of voiding dysfunction. The identification of the underlying causes of failure may improve the success rate of the urethral sphincter BoNT-A injection.

## 4. Conclusions

The urethral sphincter BoNT-A injection is effective in treating voiding dysfunction in non-SCI patients. The results of this study showed that patients with DV may benefit the most in terms of subjective and objective parameters, whereas those with DU and PRES also have a fair response in approximately half of the patients. PVR > 250 mL may indicate a poor treatment outcome in patients with DU.

## 5. Materials and Methods

This study retrospectively analyzed patients with voiding dysfunction who were refractory to medical treatment. They received 100 U of BoNT-A injection (onabotulinumtoxinA, Allergan, Irvine, CA, USA) via urethral sphincter by cystourethroscope. All patients underwent videourodynamic study (VUDS) assessments to identify the underlying etiology of the lower urinary tract dysfunction before administering BoNT-A injections. Patients with anatomical BOO of various etiologies, such as urethral stricture, bladder neck obstruction, or benign prostatic hyperplasia, were excluded from the study. SCI patients with DSD were also excluded. Finally, patients with DU who required abdominal straining for spontaneous voiding and those who required urethral sphincter non-relaxation while voiding were included in the final analysis. The study was conducted in accordance with the Declaration of Helsinki, and the protocol was approved by the Ethics Committee of Hualien Tzu Chi Hospital, Buddhist Tzu Chi Medical Foundation (IRB 111-247-B), and waived informed consent due to its retrospective nature.

The urethral BoNT-A injection treatment was performed under light intravenous general anesthesia. A total of 100 U BoNT-A was given via transurethral sphincter injections per our previous report [24]. One vial of 100 U botulinum toxin A was reconstituted with normal saline to 5 mL. Every one mL of BoNT-A solution was injected into the urethral sphincter at the 2, 4, 6, 8, and 10 o’clock positions transurethrally in men. Transcutaneous injections were administered to the urethral sphincter along the urethral lumen at the 2, 4, 6, 8, and 10 o’clock positions of the sides of the urethral meatus in women. A Foley catheter was placed overnight after BoNT-A injections and removed the next morning. Self-voiding status was recorded at the outpatient clinic. In our previous experiences, the effect of BoNT-A on the urethral sphincter’s function appeared approximately three days after injection and the maximum therapeutic effect was attained 2 weeks after treatment [24]. Three days of antibiotics were given to prevent UTIs. After BoNT-A injections, we discontinued other medication for reducing urethral resistance.

Baseline VUDS parameters, including the cystometric bladder capacity (CBC), voided volume (VV), PVR, Q_max_, first sensation of bladder filling (FSF), first desire (FS), urge sensation (US), bladder compliance, and detrusor pressure at the maximal flow rate (P_det_.Q_max),_ were recorded. VE was calculated as follows: voided volume/total bladder capacity × 100 [25]. The maximum filling volume was defined if patients consistently had no urge to void at 600mL. Bladder compliance was measured at CBC. The terminology used in this study was based on the recommendations of the International Continence Society [1]. All patients were regularly followed up at a single center, and repeated VUDS was performed within 6 months. For analysis of the treatment outcome, the urethral sphincter dysfunctions were categorized as DV and PRES, according to the electromyographic reports and pictures during voiding cystourethrography on VUDS. DV was diagnosed when high detrusor pressure, intermittent or increased external sphincter EMG activity, and a “spinning top” urethral appearance on cinefluoroscopy during voiding occurred together. On the other hand, PRES was diagnosed based on low voiding P_det_.Q_max_, with non-relaxation of urethral sphincter EMG and narrow urethral during urination.

The primary outcome of this study is the VE after treatment which was assessed after the urethral BoNT-A injection to report their global response assessment (GRA) by reviewing the patient’s chart over 1 month and graded from 0 to 3. Patients who needed indwelling urethral catheters or suprapubic cystostomy (IDC) and clean intermittent catheterization (CIC) and those with a VE of less than 33% were classified as those with treatment failure (grade 0). When patients who were able to urinate (either abdominal straining or spontaneously) with a VE of 33.3–66.7% were considered to have mild improvement (grade 1). Those who could urinate with a VE of 66.7–90% were considered to have experienced moderate improvement (grade 2). If patients could urinate with VE of 90–100% were considered to have experienced marked improvement (grade 3). Patients who could achieve grade 2 or 3 improvements after treatment were considered to have satisfactory outcomes. For recording the VE, patients were asked to urinate at a strong/urgent desire to the uroflowmetry. If voided volume plus PVR was less than 250 mL, patients were requested to urinate again. The secondary endpoint is the VUDS parameters before and 6 months after treatment. Adverse effects after BoNT-A injections were also recorded.

### Statistical Analysis 

Categorical variables were presented as frequencies (proportions), while continuous variables were expressed as the mean ± standard deviation. Urodynamic parameters at baseline and after treatment were compared using the paired *t*-test, which was also used to determine differences in symptom scores and objective parameters between groups. On the other hand, the analysis of variance was used to determine differences between subgroups. The Chi-square test was used to analyze categorical variables.

To identify the predictive factors of a good treatment outcome, we used a forward selection method to perform multivariate analyses. Receiver operating characteristic (ROC) curve analyses were performed to identify the optimum cutoff value for predicting better outcomes for DU patients. Accordingly, the optimal cutoff value was indicated by the point on the ROC curve that was closest to the upper left-hand corner. All statistical analyses were performed using SPSS for Windows (Version 16.0; SPSS, Chicago, IL, USA). A *p*-value of <0.05 was considered statistically significant.

## Figures and Tables

**Table 1:** Baseline characteristics and demographics of non-spinal cord injured patients with voiding dysfunction.

	DU (*n* = 60)	DV (*n* = 77)	PRES (*n* = 24)	Total (*n*=161)	*p*-Value
Age	60.3 ± 19.4	54.0 ± 22.5	67.0 ± 11.6	58.1 ± 20.6	0.007
Male	*n* = 19	*n* = 10	*n* = 14	*n* = 43	0.000
Female	*n* = 41	*n* = 67	*n* = 10	*n* = 118	
DM	30.0%	19.5%	29.2%	23.1%	0.320
CVA	18.3%	11.7%	8.3%	12.2%	0.379
Parkinsonism	1.7%	3.9%	8.3%	4.1%	0.309
Cervical cancer s/p radical surgery	18.3%	7.8%	4.2%	12.2%	0.013
s/p spine surgery	13.3%	3.9%	8.3%	8.2%	0.123
s/p bladder outlet surgery	78.3%	26.0%	45.8%	44.2%	0.000
Immune disease	10.0%	1.3%	16.7%	7.5%	0.007
Recurrent UTI	33.3%	18.2%	4.2%	23.8%	0.008

DU: detrusor underactivity, DV: dysfunctional voiding, PRES: poor relaxation of the external sphincter, DM: diabetes, CVA: Cerebrovascular accident, UTI: urinary tract infection.

**Table 2:** Comparison of videourodynamic parameters before and after the urethral Botox injection in non-spinal cord injured patients with voiding dysfunction.

		DU (*n* = 60)	DV (*n* = 77)	PRES (*n* = 24)	*p*-Value
FSF (mL)	Baseline	172.8 ± 80.9	105.4 ± 53.2	149.5 ± 59.0	0.068
	Follow-up	165.8 ± 88.9	121.7 ± 64.5	214.3 ± 45.8	
FS (mL)	Baseline	241.5 ± 76.8	173.9 ± 79.7	236.8 ± 89.8	0.163
	Follow-up	245.1 ± 127.4	202.9 ± 100.9	290.3 ± 55.1	
US (mL)	Baseline	303.3 ± 117.1	209.1 ± 100.9	286.0 ± 113.8	0.151
	Follow-up	286.6 ± 133.9	230.5 ± 105.5	336.4 ± 64.2	
CBC (mL)	Baseline	402.9 ± 174.3	317.9 ± 135.8	432.0 ± 115.4	0.726
	Follow-up	399.5 ± 168.5	296.7 ± 153.3	412.4 ± 173.3	
Compliance (mL/cmH_2_O)	Baseline	63.9 ± 96.5	40.4 ± 62.5	53.8 ± 25.8	0.186
	Follow-up	47.4 ± 36.7	59.6 ± 71.5	52.3 ± 19.5	
P_det_.Q_max_ (cmH_2_O)	Male (BL)	10.3 ± 11.6	34.00 ± 15.4	32.0 ± 13.1	0.584
	Follow-up	19.3 ± 25.6	31.1 ± 17.6	27.6 ± 14.6	
	Female (BL)	5.69 ± 8.09	60.1 ± 36.0	74.3 ± 90.0	0.000
	Follow-up	4.89 ± 8.76	47.6 ± 32.6 *	13.3 ± 7.37	
Qmax (mL/s)	Baseline	2.70 ± 4.41	6.54 ± 5.06	8.29 ± 5.96	0.987
	Follow-up	3.44 ± 5.31	7.11 ± 5.58	8.71 ± 6.13	
Volume (mL)	Baseline	60.5 ± 115.7	131.1 ± 117.3	219.1 ± 142.5	0.961
	Follow-up	73.1 ± 130.6	139.3 ± 131.4	214.4 ± 163.7	
PVR (mL)	Baseline	344.4 ± 206.8	183.1 ± 143.8	198.3 ± 160.3	0.680
	Follow-up	326.5 ± 197.1	195.3 ± 147.8	231.0 ± 176.8	
VE	Baseline	0.16 ± 0.29	0.41 ± 0.34	0.52 ± 0.38	0.811
	Follow-up	0.18 ± 0.32	0.38 ± 0.36	0.58 ± 0.41	
BOOI	Male (BL)	8.07 ± 9.21	16.0 ± 13.6	13.5 ± 19.2	0.580
	Follow-up	15.0 ± 24.9	10.1 ± 14.8	9.75 ± 21.6	
	Female (BL)	−1.51 ± 12.4	48.2 ± 35.8	53.6 ± 99.4	0.002
	Follow-up	−2.83 ± 14.2	30.0 ± 33.5 *	−12.6 ± 19.0	

FSF: first sensation of filling; FS: full sensation; US: urge sensation; P_det_: detrusor pressure; Q_max_: maximum flow rate; Vol: voided volume; PVR: post-void residual; CBC: cystometric bladder capacity; VE: voiding efficiency; BOOI: bladder outlet obstruction index. P, comparison of the changes in variables from baseline and after treatment among each group. * *p* value < 0.05 comparison between baseline and after treatment.

**Table 3:** Treatment outcome per the scaled Global Response Assessment (GRA) after urethral Botulinum toxin A injections.

		GRA = 0–1 (*n* = 61)	GRA = 2 (*n* = 69)	GRA = 3 (*n* = 31)	*p*-Value
Age (years)		64.3 ± 17.1	54.2 ± 22.5	58.2 ± 18.2	0.016
Gender	Male	21 (48.8%)	13 (30.2%)	9 (20.1%)	0.127
	Female	40 (33.9%)	56 (47.5%)	22 (18.6%)	
Voiding dysfunction	DU	30 (50.0%)	25 (41.7%)	5 (8.3%)	0.002
	DV	18 (23.4%)	38 (49.4%)	21 (27.8%)	
	PRES	13 (54.2%)	6 (25%)	5 (20.8%)	

DU: detrusor underactivity, DV: dysfunctional voiding, PRES: poor relaxation of the external sphincter.

**Table 4:** Multivariate analysis of factors associated with a global response assessment ≥2 in non-spinal cord injured voiding dysfunction.

Variables	Odd Ratio (95% CI)	*p*-Value
DV	3.630 (1.617–8.152)	0.002
Voided volume	1.004 (1.001–1.008)	0.014
Cervical Cancer s/p radical surgery	0.290 (0.092–0.909)	0.034
Recurrent UTI	3.949 (1.453–10.732)	0.007

DV: dysfunctional voiding, s/p: status post operation, UTI: urinary tract infection.
